# American Medico-Psychological Association—Sixty-first Annual Session, San Antonio, Texas, April 18, 19, 20, 1905

**Published:** 1905-06

**Authors:** 


### Society Notes.

### American Medico-Psychological Association—Sixty-first Annual Session, San Antonio, Texas, April 18, 19, 20, 1905.

[From the *Express.*]

The most exclusive organization of medical scientists in the United States is in session in this city at the Menger Hotel. The present meeting of the American Medico-Psychological Association is the sixty-first in its history, one having been held each year since the organization of the society in 1844 under the name of the "Association of Medical Superintendents of American Institutions for the Insane." There are 403 members of the Association. The number in attendance at the session yesterday was sixty-three.

All of the addresses of welcome delivered on behalf of the State, city and county medical associations were eloquent, with real hospitality and the zeal of enthusiasm in the words spoken. The visitors appreciated the warmth of welcome accorded them, and at the beginning of the afternoon session adopted by unanimous vote a resolution thanking the speakers who made them welcome to this city.

Dr. F. E. Daniel, President of the State Medical Association, discussed the various propositions that are of interest to alienists. He urged that jurisprudence has been falling behind science in the various matters wherein they are co-related. Science recognizes forty-two forms of mental disease, while jurisprudence only recognizes two. The jury system had some bouquets of a doubtful character tossed to it by Dr. Daniel.

The welcome by Rev. Homer T. Wilson was happy in style, and made a hit with the visitors and home people alike. It was witty and light and local in color.

The address of F. C. Davis, city attorney, on behalf of the city, was characteristically modest and warm. It made the visitors feel at home.

Dr. L. L. Shropshire, for the Bexar County Medical Society, made the visitors welcome in a happy and graceful manner. He discussed matters of interest in the course of his brief remarks and showed the visiting doctors a whole-souled and gratifying happiness at their presence.

Dr. Graves, Chairman Committee of Arrangements, said:

*Mr. President, Ladies and Gentlemen:*

Texas will extend to her friends from the East and the West, the North and the South, a cordial greeting today, and I introduce to you first, a gentleman who has grown somewhat gray of head in fighting the battles of organized, legitimate medicine, but whose heart, like that of Dr. Oliver Wendell Holmes while living, is still young, and whose mental faculties, active and alert, present that proper combination of maturity and wisdom with the sparkle and vigor of youth. I have the pleasure of introducing to you Dr. F.

E. Daniel, President of the State Medical Association, who will welcome you for the three thousand doctors of Texas. (Applause.)

Dr. Daniel:

*Mr. President and Members of the American Medico-Psychological Association, Ladies and Gentlemen:*

It is my privilege, my great pleasure and a distinguished honor to welcome you in the name of my colleagues of the State Medical Association; and on their behalf I extend to you the right hand and greet you, friends and brothers, and welcome you to this Empire of the West. Texas, though only a younger member of the great sisterhood of States, is famous the world over. Who, in the remotest part of the civilized world, does not know of the Lone Star of Texas—of the Battle of the Alamo? We are a happy, prosperous people—proud of our State, proud of her splendid citizenship, of her history—proud of her institutions, her great wealth and wonderful resources, as yet scarcely touched. We are proud of her strong men and beautiful women, and proud of our State institutions. Especially are we proud of our splendid psychopathic hospitals and their talented superintendents, who are an honor to the State; and doubly proud of the splendid provision the State has made for the care of the unfortunates.

We welcome you to the famous city of the Alamo. So long as civilization shall endure, the names of Travis, Crockett, and Bowie will live in song and story, and the tale of the Alamo will sound down the ages to the remotest time, along with that of Marathon and Thermopylæ. Here was poured out that "boon that heaven holds dear,—

> "The last libation that Liberty draws,
> From hearts that bleed and break in her cause."

As from Daphne's virgin blood sprang the laurel,—the emblem of honor and immortality,—so the blood of our hero patriots enriched the soil of Texas and became the fruitful germ whence has grown a magnificent civilization who will ever honor their names and keep their memory green.

Many of you gentlemen, perhaps, have not visited Texas before. You have had no experience in the delights of a sun-kiss't, blossom-blest land like this. Your visit is as timely as it is welcome. You see the land at its best. You see the old Spanish city in her Easter attire, as gay and happy and blushing as a bride. Cybele has unlocked the store-house of her treasures and scattered her jewels broadcast over the land. Our boundless, billowy prairies,

"as broad as the sweep of the tidal wave's measureless motion," were, a little while ago, the home of the buffalo and the hunting ground of the savage. Today they are brilliant with the bloom and fragrant with the perfume of the lily and the rose, as well as the wild fox-glove and the gentian, the bluebells and the daisy. Dotted with flocks and herds and villages and hamlets and happy homes, the air is vocal with the hum of husbandry! The cheery "gee-haw" of the plowman is everywhere heard, as he prepares for that harvest that feeds and clothes the world; and as the mellow mould turns from his plow, see the eager, noisy flocks of blackbirds following in his wake, to catch the "early worm."

"The down of the thistle and the bob-o-link's whistle
Are blent with the vernal day's light and perfume."

In our cities, too, the rattle of machinery, the noise of traffic, and the hum of industry are sweet, familiar sounds. Across the State, from Texarkana to Brownsville, and from Longveiw to El Paso, from Denison to the gulf, the State is ribbed with iron bands, and over broad plains and mountain steeps and mighty rivers, and purling brooks and hill and dale, lumbering trains bear to the bosom of the ocean the products of our soil. It is a pretty sight to stand on Chautauqua hill at San Marcos, the "loveliest village of the plain'" and watch two splendid vestibule trains—the I. & G. N. "Flyer" and the "Katy" "Cannon Ball"—racing in sight of each other for miles, to the quaint old city, laden with tourists to see the Alamo and the long line of missions, founded two hundred years ago. One can but stand and wonder at the durability and beauty of the Moorish architecture, and towers and minarets of two centuries ago, and the quaintly-carved doors and altars, the work of the fanatical Franciscan Monks; and bearing health-seekers to bask in our soft, Southern sun and to breathe the balm of our fragrant air, and drink in deep draughts of health. They are welcome. We dwell, indeed, in a favored land. The setting sun lingers to throw a last kiss "good-night" to Texas, and sinks to rest suffused in a halo of his own blushes. This is a land of the pomegranate and the fig, the magnolia and the olive:

"Here rage no storms; the sun diffuses here
His tempered beams thro' skies forever fair;
Here gentle airs o'er brakes of myrtle blow,—
Hills greener rise and purer waters flow.
Here bud the woodbine and the jas'mine pale,
And every bloom that scents the morning gale,

While thousand melting sounds the breezes bear
In silken dalliance to the dreaming ear,—
And golden fruits mid shadowy blossom glow."

But gentlemen, you are not here alone for pleasure. You are not here for the gratification of the senses, however delightful. You are here to work for humanity and the advancement of that young science, born but yesterday, of which you are the distinguished and honored exponents and apostles. There is no brighter page in the history of medicine than that wherein is recorded the birth, growth and evolution of psychiatry,—the scientific treatment of the diseased mind. It makes us shudder to look back and recall the cruelty and barbarity inflicted through ignorance upon the unfortunates, even one hundred years ago. It makes us sad to recall what was their fate before God inspired Pinel and Esquirol and Ray, and Rush, and Galt, and Stribling, and Woodward, and Brice—and that angel of mercy, ever blessed be her name, Dorothy Dix,—through all of whose inspired and divinely-guided labors we have reached the position of our splendid psychopathic hospitals and the rational treatment of diseases of the mind. High up on Fame's proud temple their names are inscribed in letters of living light and will endure forever!

It makes us dizzy to recall the advances that have been made in the last quarter of a century, for within that time the new psychology has been born. It was not evolved out of the old psychology, for that was such in name only. The immortal Locke, the father of the old psychology, had not, of course, the faintest conception of the wonderful truths and discoveries that have occurred, which show the mind in all of its multitudinous phases of ideation, thought, memory, and consciousness itself, to be the function of the brain, evolved by chemical action,—a force, a mode of motion, if you please, specialized energy, transformed by the original dynamo, the brain,—into what I believe is identical with electro-magnetic energy..

Locke and his followers of the old school, and all the world conceived of the mind as being the soul; something outside of, external to, independent of the organism, and which entered the body of the babe at birth, controlled the life of the man, shaped his destiny, and which at death was liberated and was immortal. That was a psychology of speculative philosophy,—the "science of the soul" (*psucho*). We have now a new psychology, — Professor James to the contrary, notwithstanding,—a science of the mind.

All scientific men now recognize that the mind is purely a physiological process, independent of any external or supernatural causes.

If it makes us dizzy to contemplate the advances of psychology in the past, it makes our heads swim to contemplate the possibilities of the future. We stand today, gentlemen, upon the shoulders of our immediate predecessors, and as we look into the opening vista of the twentieth century, the field opens, ever broader and unending, and we are startled at the possibilities that await the further investigation of the brain cells. While much has been accomplished, there is much yet to be accomplished. Surely, it was thought that when Flechsig demonstrated the thought cells, and the sensory and motor areas were clearly mapped out, we had reached the limit; but there are other problems. What is consciousness? How does it arise? Given the thought cells, how is thought produced? How is it distilled from the elements of the food that float in the blood that bathes every other cell in the body?

But, gentlemen, you are less concerned with psychology than with psychopathology and psychiatry. You are here to study your special branch of science and to devise means and methods for the better construction and management of your institutions, and the care of your patients; but we have well-nigh reached perfection in that. You deal with the sick mind. It is yours to

"* * * minister to a mind diseased.
To pluck from the memory a rooted sorrow;
To raze out the written troubles of the brain,
And with some sweet, oblivious antidote
Cleanse the stuff'd bosom of the perilous stuff
That weighs upon the heart."

There is much work before you. You, gentlemen, in your organized capacity, are an immense power for good. That power should be exerted. While science has advanced along this line, jurisprudence has stood still, and I say that it is a disgrace to the State of Texas that, while forty-two or forty-four forms of insanity are recognized by alienists, jurisprudence recognizes only two forms, the "natural" and the "acquired," and the absurd test of knowing right from wrong, adopted in the McNaughton case two hundred years ago, is still the rule in our insanity trials. Unfortunates who are arraigned on a charge of murder, and where insanity is the defense, have not the benefit of a diagnosis by the light of modern science.

Much could be said along this line. I would refer to the absurdity of having a jury of laymen to decide such a question. In our jury system, the more ignorant a man is, the better qualified he is to act, according to the practice of our jurisprudence. Experts testify, and they always differ, and the courts call in twelve farmers, mechanics, tinkers, and tailors, and candlestick makers, day laborers, and what not, to decide.

But, gentlemen, I am occupying your time. There are many things you will have to consider. The jurisprudence of insanity has not progressed with science. You should have influence with Congress, but I am afraid, in many of the States, you will find that the most of the legislators ignore science, and are moved solely by sentiment. There is one problem before you to which I wish to call attention. I refer to the alarming, terrible increase of insanity. What is the cause of it? Dr. Graves, in his admirable address delivered at Galveston last month, makes the startling assertion that the insane in Texas in the last forty-five years have increased 6800 per cent, while the population has increased but 504 per cent; that is nearly 14 to 1. What is the cause of it? How can it be arrested? It is out of all proportion to the population and out of all proportion to the increase of crime which is truly appalling. Something must be done, if possible. And it does seem to me, gentlemen, that our whole humanitarian system aims at and fosters the survival of the unfit, and the propagation of the defectives. It is race suicide.

And now again, welcome. We set before you bread and salt—symbols of our most generous hospitality. We would send you home with a song in your hearts, and ask for ourselves a place there. We would say, "Rest you here; bide with us awhile. We would do you honor."

### PRESIDENT'S RESPONSE TO WELCOME.

President Burgess, of Canada, in response to the various addresses of welcome, said:

We have long heard of your great State, and rejoice to be now able to see it for ourselves. I say great State, for assuredly it is great. Great in that it is the largest State in the Union, exceeding in size the whole of New England with Maryland, New York, Ohio, Pennsylvania and the two Virginias thrown in; great in that it has a larger number of miles of railway than any other State; great in that it is the largest producer of cattle and cotton and stands fifth of the States for population, with the prospect of outstripping all of them in this also. Nor is it only in these respects that it is great. What State can boast of older or prouder historical reminiscences? Over it has floated no less than six different flags, to each of which is attached a host of soul-stirring memories; those of France, Spain, Mexico, the Texan Republic, the Southern Confederacy and the United States. On its soil was fired the last shot of the great Civil War, and in its capital stands a monument bearing one of the noblest epitaphs ever penned—"Thermopylæ had its messenger of death, the Alamo had none."

Though many of you may not be aware of it, there is a very close bond of union between the State of Texas and our Canadian province of Quebec, in that both are indissolubly linked with a name dear to all Canadians, that of the intrepid explorer, Robert de la Salle. Somewhere in Texas soil were interred the remains of that great and heroic man, buried by his faithful adherent, Father Douay, after his foul murder by his mutinous followers, and less than three miles from the hospital over which I have the honor to preside are the ruins of La Salle's Canadian home, the home which he built and in which he lived for some four years of his early Canadian life, the home in which were planned the great schemes for the extension of new France, which engrossed his remaining days.

Not less rejoiced are we that in visiting your State for the first time, our meeting place should be the ancient and beautiful city San Antonio, the birthplace of Texas liberty, the scene of heroic deeds, the place where Travis, Crockett, Bowie and all their valiant band fought their last fight and gave their lives that Texas might be free.

But, gentlemen, it is not alone as sight-seers and hero worshippers we are here. We have in addition a serious purpose in view; the discussion of problems connected with our particular branch of medicine, and the practical matters associated therewith. The noble and Christ-like work of ministering to the mind diseased, in which we are engaged will, we trust, receive an added impetus from our meeting in your midst, and the close of our sessions will, I feel assured, see each of us the better prepared and the more resolute to devote himself with all his strength to the tasks assigned him. There is much, very much, to be done in our speciality, and I for one rejoice that it is so, with all my heart saying:

God be thanked that the dead have left still
Good undone for the living to do—

Still some aim for the heart and the will
And the soul of man to pursue.

Again I thank you for your cordial welcome. We came as strangers, but feel so no longer. You have already made us feel that we are at home, and that only friends surround us.

[The other addresses were not reported.—Ed.]

The forenoon session of the Association was closed by the reading of a paper prepared by Dr. Robert C. Kemp, of New York, on the "Relation of the Gastro-intestinal Tract and Mental Diseases."

### CAUSES OF INSANITY.

The feature of the first day of the meeting of the American Medico-Psychological Association in this city Tuesday was the annual address of the President of the Association, Dr. T. J. W. Burgess, of the Hospital for Insane, Montreal, Canada. Dr. Burgess discussed the history and condition of the insane asylums of Canada. His paper began with the beginning of the care of mental unfortunates in the early days of the Dominion and came down to the present, and the troubles of the present in the prevention, cure and care of insanity.

Dr. Burgess showed that insanity is increasing in Canada, as elsewhere on this Continent. He attributes this increase to three general causes; heredity, the stress of modern life, and unrestricted immigration. But of these causes, heridity is the chief and overawing factor in the increase of the world's unfortunates. While the people are averse to giving out facts in regard to mental obliquity in their families, and accurate statistics are difficult of obtaining, it is believed that the rate of increase in mental disorders is so great as to call for stringent measures to suppress it. The line of argument whereby people come to think that mental disease is a disgrace and thus seek to suppress the information that it exists in their families is not sound, and the people should be educated to understand that mental diseases are no more disgraceful nor to be hidden than physical diseases.

Prevention of insanity is believed to be possible only by the prevention of union and reproduction of persons afflicted with mental diseases. This should be accomplished by laws directed to that end.

The session Wednesday was directed to hearing the annual address, an able paper on brain surgery, election of new officers, and discussion of the papers and routine business matters. In the afternoon a drive was taken to the missions.

Dr. A. E. McDonald, of New York, made reports on several matters and talked at some length on the relation of the Medico-Psychological Association with other prominent medical societies. Upon motion the Association unanimously decided to appoint Dr. A. E. McDonald delegate to the International Medical Congress at Lisbon in 1906. Upon invitation the delegate will present a paper before the International Society.

### NEW OFFICERS ELECTED.

The report of the nominating committee was read by Dr. Charles W. Pilgrim. It was adopted as read. It provides for the following officers of the Association for the ensuing year: President, C. B. Burr, Flint, Mich.; Vice-President, C. G. Hill, Baltimore, Md.; Secretary and Treasurer, E. C. Dent, New York; Councilors, G. A. Smith, New York; W. F. Beutler, Wisconsin; J. T. Searcy, Alabama; W. H. Beemer, Canada; M. L. Parry; Kansas; Auditors, A. W. Hurd, New York, and W. H. Hancker, Delaware.

St. Paul, Minn., was selected as the next place of meeting of the Association. The date will be fixed by the Committee on Arrangements, who are H. A. Tomlinson and A. F. Kilbourne, both of Minnesota.

### DR. SEARCY ON TRIPARTITE MENTALITY.

Dr. James T. Searcy, of Tuscaloosa, Ala., Thursday presented to the Medico-Psychological Association his annual address. It was a stately and studied paper on the subject of the mind. The title given his paper by Dr. Searcy is "Tripartite Mentality." Dr. Searcy showed the triple functions of the mind and the relation of each function to the others. He divided the proposition into three methods of managing information—receiving, adjusting and emitting. He first divided mentality into normal and abnormal. He showed that abnormality, however slight, is a phychopathic condition, but that abnormal minds are not insanity until they reach that stage of abnormality which comes within the cognizance of the law.

Dr. Searcy held to his tripartite mentality proposition through the entire course of his paper. He touched upon the old man question in his remarks, and the suggestion seemed to bring to his hearers a thought of the Dr. Osler proposition that old men are not much account anyway, when some point was made to the relative disparagement of the old man in some lines of thought and action.

In the division of the subject into the three parts, learning, judgment and execution, Dr. Searcy made the point that anybody can learn, the younger men, of course, learning more quickly, but all being able to receive impressions. But under the heads of judgment and execution the average man is weak. Dr. Searcy here made a point for the horse-sense fellows by stating that common sense is born in a man, not taught to him.

SURGERY FOR RELIEF OF INSANE.

The paper by Dr. M. E. Witte, medical superintendent of the Hospital for the Insane at Clarinda, Iowa, who is one of the most noted surgeons of the country, was on "Surgery for Relief of the Insane Condition."

The paper by Dr. Witte received the undivided attention of the Association. It was profound and voiced a sentiment that was general in the membership of the Association who participated in the discussion opposing brain surgery, save in case of injury received immediately prior to the surgical operation.

The papers and discussions hinged about the proposition to prevent insanity by eliminating the possibility of reproduction of the affected by legislative enactment. The formation of public opinion to that end was advocated.

Interest in yesterday's sessions centered in the paper read by Dr. H. D. Miller, of Taunton, Mass., and the discussions that it brought out. The subject of the paper was "Huntingdon's Chorea." The paper gave a minute description of the family history of a case in the Taunton Hospital, and showed the awesome spread of the mental malady from one case. In the case described twenty-seven descendants of one ancestor were found to be affected to a greater or less degree. Not one of them escaped the mental taint. The description of the cases and their development showed that none had escaped the curse of the insane ancestor, and that in one or more instances the woman to whom the patient had been married was in ignorance of the family history, and not aware of the prospect of insanity, as held out in the life of the man she married. The peculiar disease known as Huntingdon's Chorea develops after the third decade in the life of the descendant of the victim of the disease. It never fails to develop, Dr. Miller shows, although it sometimes is not serious. The mental symptoms are not so obvious as the physical evidences of the approach of the malady of mind. There are rare insances of this chorea developing *de novo.*

Dr. Miller drew the conclusion that the State should control the marriage of persons afflicted with mind maladies, and thus rob the disease of some of its terrors.

Dr. Charles B. Hill, of Baltimore, Md., who was yesterday elected to be Vice-President of the Medico-Psychological Association, and who will consequently be elected to the presidency one year hence, is one of the interesting men of the Association. He is among the most prominent alienists in the United States, and is frequently called to testify in cases involving the mental fitness of parties in court. Dr. Hill presented a paper on the "Relation of Mental and Nervous Diseases of the Liver." He pointed out a great many things in his introduction. The rest of the paper was highly technical, and drew encomiums from the assembly at its close. But in getting started the doctor discussed alcohol as it affects the liver. He showed from the recital of experiments on guinea pigs that alcohol, whisky and absinthe are not nearly so damaging to the liver as has been made out. He related the story of a friend of his who was bibulously inclined, and when taking a drink had the habit of remarking that he would close one more cell in his liver and then quit.

Dr. A. W. Hurd, of Buffalo, N. Y., read a highly technical paper on "Korsakoff's Psychosis." This paper showed that in most cases the toxic agent is alcohol.

Dr. John Punton, of Kansas City, presented an interesting paper on "Mysophobia," and detailed the story of the degenerate whose hallucination was cleanliness. The papers discussed neurasthenia and differentiated between obsessions, besetments and fixed ideas.

A paper by Dr. Charles K. Mills, who is one of the greatest living authorities on the brain, its structure, functions, and diseases, was read by Dr. B. M. Caples, of Wisconsin. The paper was on "Crossed Ambliopia with Vascular and Degenerate Lesions."

A paper on "Surgical Cases in Manhattan State Hospital, East," was read by J. P. Wade, of Baltimore. It had been prepared by Dr. John B. Knapp.

A paper by Dr. LeRoy Broun on "Gynecological Surgery in Manhattan State Hospital, West." Dr. Broun is a noted surgeon in women's pelvic troubles. The discussion of the paper hinged upon the proposition that it is a good thing to treat insane patients just as sane ones should be treated.

An interesting paper on "Tuberculosis Among Insane," prepared by Dr. C. F. Haviland, was read by R. H. Hutchings, of Ogdensburg, N. Y. It was the theory of the author of the paper that insane cases *de novo* are so largely contributed to by alcohol and syphilis as to lead to the conclusion that tuberculosis is general and exists in most cases of insanity; for consumption is fostered by alcohol and syphilis. The advisability of isolation in tents and the tent system for out-door treatment of consumptives was advocated, and the discussion developed that the out-door method in any climate is considered the best.

One interesting and pleasant feature of the day's business was the appearance before the Association as a speaker of Dr. D. R. Wallace, an aged physician of Waco, Texas, who spoke briefly to the assembly and recited matters that are not within the memory of even the older members of the Association. Dr. Wallace was on the program to deliver a paper on "Expert Testimony of the Doctor in Court," but he rather talked of the progress that has been made in medical science, and in the Association of which he was a member thirty-one years ago. Dr. Wallace was one time Superintendent of the Texas State Lunatic Asylum and also of the North Texas Hospital for the Insane. His name was proposed for honorary membership and he was unanimously elected, and will be a member for the rest of his life. Dr. Wallace is 80 year of age, and is hale and active and looks much less than that age.

Delegates to the Medico-Psychological Association convention were banqueted at Turner Hall Thursday night. The menu was Mexican, and to many of them was novel.

These men of science sat around the banquet board and partook of the fiery viands prepared for them with a relish that was pleasing to note. For the nonce they forgot the niceties of their science. Nerve centers and brain cells were relegated. Wit, fired with pepper, and wisdom soothed and cooled with beer, prevailed. The speeches were cast in a semi-serious vein. For the most part they were humorous, the banqueters eschewing anything smacking of the scientific. They gave themselves over to good-natured raillery.

President W. L. Stiles of the Business Men's Club was toastmaster. Covers were laid for 125 guests. The menu was as follows:

Chile Con Carne.

Sopa de Arroz. Frijoles.

Tortillas.

Cerveza.

---

Mole Poblano.

Ensalada de Aquacarte.

Tamales.

---

Chiles Rellenos.

---

Pina.

Cigaros. Cigaritos.

Cafe.

"Beer" *ad libitum.*

Dr. T. J. Burgess, of Canada, was the first speaker introduced, being President of the Association. Dr. Burgess made a happy speech, in which he was highly complimentary to Texas and San Antonio. He had heard a story to the effect that it was so hot in Texas that when a man died it made very little difference where his soul lodged, so far as comfort was concerned. He had not found it so. He had heard of hold-ups and men carrying guns. He had not been held up, even at the bar, and had not seen anything resembling a cowboy or a rifle.

"Texas is an ideal State," he said, "and if I had to choose a place to live the rest of my days I would pass them in San Antonio," he said in conclusion. The sentiment was enthusiastically applauded.

Dan G. Gillette was introduced as the "Dean of the Business Men's Club." He said he faced a community of specialists with trepidation.

"I woke the other morning to find that a man's usefulness is at an end at 46 years," said Mr. Gillette. "So, chronologically, I should have been chloroformed three years ago," which evoked laughter. He said in Boston a man could have any kind of a disease and believe anything he pleased and always find a specialist to help him along.

"While you are in Texas," continued Mr. Gillette, with a serious expression, "I hope you will discover the bacillus of Texas politics. (Laughter.) I would suggest that your Association send a committee to Boston to get bacillus, because I understand it abounds there. You could return to Austin and find thousands of patients, as I am told there is every kind of political insanity dreamed of there." Adverting to the dinner, Mr. Gillette reminded them that if any one of them should die on the prairie they would not be bothered by the coyote.

"If you are subject to gout," he said, "I would advise you to prepare your strenuous vocabulary in advance. I remember swearing in four different languages on my first experience, and regretted that I did not know ten more."

The toastmaster, President of the Club, escorted Mrs. Dr. Daniel to a seat beside him at the head of the table. Proposing a toast to "Woman," he called on Dr. Daniel to respond, and said: "Dr. Daniel is famous for many things, but he is more distinguished as the *husband of Mrs. Daniel* than for anything else."

The *Express* says: Dr. F. E. Daniel, of Austin, President of the State Medical Association, responded to the toast, "Woman." He met the delicate task in an admirable manner. He said he belonged to the regime of the Old South whose men placed woman on a pedestal and bowed in worship at her feet. It has been said that an honest man is the noblest work of God. He considered a noble woman the honest work of God. He recalled what Ambassador Choate had said at a London dinner when he was asked what he had rather be of all things if he had the privilege of exchanging his present high station for anything of his choice. With a graceful bow the ambassador turned to his wife and said, "Mrs. Choate's second husband." The speaker would say the same of Mrs. Daniel.

Charles M. Tobin made a witty speech on "The Versatility of Doctors," in which he told some stories on local physicians.

Dr. Charles G. Hill, of Baltimore, responded to the toast "San Antonio as a Meeting Place." He said he was one of the committee which selected San Antonio, and if placed on the same committee again and the matter was left to him he would have the next meeting here. After warmly expressing the sentiment of the visitors for the hospitable reception they had received, he paid an eloquent tribute to the heroes of the Alamo, concluding with the words: "Their bones are dust, their blades are rust, their souls are with God, we trust. We honor, respect and love the people who make up the citizenship of Texas," he said. He told a story of two veterans of the Civil War who were discussing the merits of the commanders on both sides. The Confederate soldier finally paid a tribute to Stonewall Jackson, who, he asserted, was now in Heaven. The Union soldier took exception to this, saying that Jackson killed too many men on the other side to ever get to Heaven. The old rebel stoutly held to his statement, and closed the argument by saying that if "Stonewall Jackson wanted to go to Heaven, all Hell couldn't keep him out." "Now, my friends, if ever I have an opportunty of coming to San Antonio at all—ah, I beg your pardon—nothing can keep me away."

Dr. John Preston, of Abilene, Texas, Superintendent of the Texas Colony of Epileptics, recited a humerous versification of the theme, "How the Fits Came Back."

A TENDERFOOT WITH A TENDER HEART.

Dr. A. E. McDonald, of New York, the eminent criminologist, was asked to discuss "The Cinnamon Bear," and talked about every other thing instead. His facetious remarks were directed towards Dr. Burgess and others. In a more serious, but still humorous vein, he concluded by saying: "We came among you as tenderfeet or tenderfoots, which ever is correct, and after hearing some of your orators we feel somewhat like tenderheads. But what I want to say is that when I recall this occasion it will be with tender heart for San Antonio and Texas."

Dr. Frank Paschal spoke on "Organized Medicine," tracing the history of the Texas Medical Association. He was followed in short speeches by Dr. M. J. Bliem, Dr. T. T. Jackson, Dr. Woodson of St. Joseph, Mo., and Dr. A. M. Hurd, of Buffalo, N. Y., and Superintendent M. L. Graves of the Southwestern Insane Hospital, Chairman Committee of Arrangements.

The usual votes of thanks were adopted. The musicale and dance at the Southwestern Insane Asylum was swell and delightful. Mrs. Dr. Frank Paschal entertained the ladies of the delegates (of whom there were about twenty-five present, of the most pleasing and charming women I ever met) with a carriage drive over the city and a reception and lunch at her elegant home. The entire party of delegates, wives and visitors were taken a tally-ho drive to the missions, the breweries, Brackenridge Park, Dr. Moody's Sanitarium, the Southwestern Insane Asylum and other points of interest.

The occasion will forever live in the memory of all who were present. Spring was in full bloom, and the air fragrant with perfume and vocal with bird song; while, at the home of many of the visitors, it was snowing!

I recall with much pleasure Dr. and Mrs. Drewry, of Petersburg, Va.; Dr. and Mrs. Applegate, of Iowa; Dr. and Mrs. Hutchings, of Ogdensburg, N. Y. (the little "Georgia girl" who drove one of the tally-ho four-in-hands, and won the admiration and special regard of all the party—a general favorite): Dr. and Mrs. John Punton, of Kansas City; Dr. and Mrs. E. C. Dent, of New York; Dr. and Mrs. B. M. Caples, of Waukesha: Dr. and Mrs. W. R. Smith, of New York; Dr. and Mrs. Crumbacker, of Michigan; Dr. and Mrs. White of Iowa; Dr. and Mrs. M. L. Graves, of San Antonio, and Dr. and Mrs. J. T. Turner, of Terrell, Texas.

The entire party made an excursion to Mexico. Much credit for the success and pleasure of the meeting is due Dr. M. L. Graves, Superintendent of the Southwestern Insane Asylum, Chairman of the Committee of Arrangements, and his associates, Drs. Worsham of the Austin Asylum, Dr. Turner of the North Texas Hospital for the Insane, and Dr. Moody, Dr. Graves' first assistant, now in charge of a private asylum of his own.

---

BRAZOS VALLEY MEDICAL ASSOCIATION.—The Brazos Valley Medical Association met at Cameron on May 9th and 10th; Dr. E. N. Shaw, President. The following papers were presented: "Pelvic Abscess," Dr. J. P. Oliver, Caldwell; "Some Additional Remarks on the Application of Plaster of Paris," Dr. W. W. Greer, Cameron; "Some Practical Points on the Use of the Microscope," Dr. J. L. Denson, Cameron; "The Value of Antistreptococcic Serum in the Treatment of Streptococcic Infection of the Lung," Dr. I. P. Sessions, Rockdale; "The Benefits and Necessity of Medical Societies," Dr. D. Monroe, Cameron. The following officers were elected: Dr. H. T. Coulter, President, Rockdale; Dr. I. N. Mayfield, First Vice-President, Deanville; Dr. H. W. Cummings, Second Vice-President, Hearne; Dr. W. B. Briggs, Secretary-Treasurer, Easterly.

